# Intussusception caused by metastatic renal cell carcinoma at the duodenojejunal junction 41 years after nephrectomy: a case report

**DOI:** 10.1097/RC9.0000000000000657

**Published:** 2026-07-07

**Authors:** Shingo Seo, Akira Nakashima, Ryutaro Sakabe, Naohiro Uraoka, Kazuhiro Sentani, Ko Tahara

**Affiliations:** a Department of Surgery, Kure Kyosai Hospital, Kure, Hiroshima, Japan; b Department of Pathology, Kure Kyosai Hospital, Kure, Hiroshima, Japan

**Keywords:** case report, duodenojejunal junction, intussusception, late recurrence, renal cell carcinoma, small-bowel metastasis

## Abstract

**Introduction and importance:**

Renal cell carcinoma (RCC) is known for its potential for late recurrence; however, ultra-late recurrence occurring more than four decades after nephrectomy is extremely rare. Small-bowel metastasis causing intussusception is also uncommon.

**Case presentation:**

A 73-year-old man underwent right nephrectomy for RCC 41 years earlier. He was referred to our hospital because of rapidly worsening glycemic control. Contrast-enhanced computed tomography (CT) revealed two hypervascular pancreatic tumors and a hypervascular small-bowel mass near the ligament of Treitz. Multiple distant metastases from recurrent RCC were suspected. Endoscopic ultrasound-guided fine-needle aspiration and endoscopic biopsy were inconclusive. One week later, the patient developed abdominal pain and vomiting. Repeat CT revealed intussusception of the proximal jejunum caused by the small-bowel tumor, requiring surgical intervention. Partial resection of the duodenojejunal junction with bypass procedures was performed. Histopathological and immunohistochemical examination revealed metastatic RCC.

**Clinical discussion:**

RCC may recur even decades after nephrectomy, and atypical metastatic sites such as the small intestine should be considered. Adult intussusception is rare and often associated with an underlying malignant lesion.

**Conclusion:**

This case highlights that RCC can recur as small-bowel metastasis even more than four decades after nephrectomy and may present as intussusception.

## Introduction

Renal cell carcinoma (RCC) is characterized by its potential for late recurrence, even more than 10 years after curative nephrectomy^[^1,2^]^. The most common metastatic sites include the lungs, bones, liver, and brain^[^3,4^]^; however, small bowel metastases are rare[5]. We report a unique case of intussusception caused by metastatic RCC in the ascending portion of the duodenum, occurring 41 years after nephrectomy and requiring surgical intervention. This case report has been reported in line with the SCARE checklist[6].

HIGHLIGHTS

- Ultra-late recurrence of renal cell carcinoma (RCC) occurring 41 years after nephrectomy.
- Small-bowel metastasis from RCC is rare.
- Adult intussusception is often associated with an underlying malignant lesion.
- Intussusception may occur due to small-bowel metastasis from RCC.
- Extremely late recurrence of RCC should be considered even decades after initial treatment.

## Case presentation

**Figure 1.**
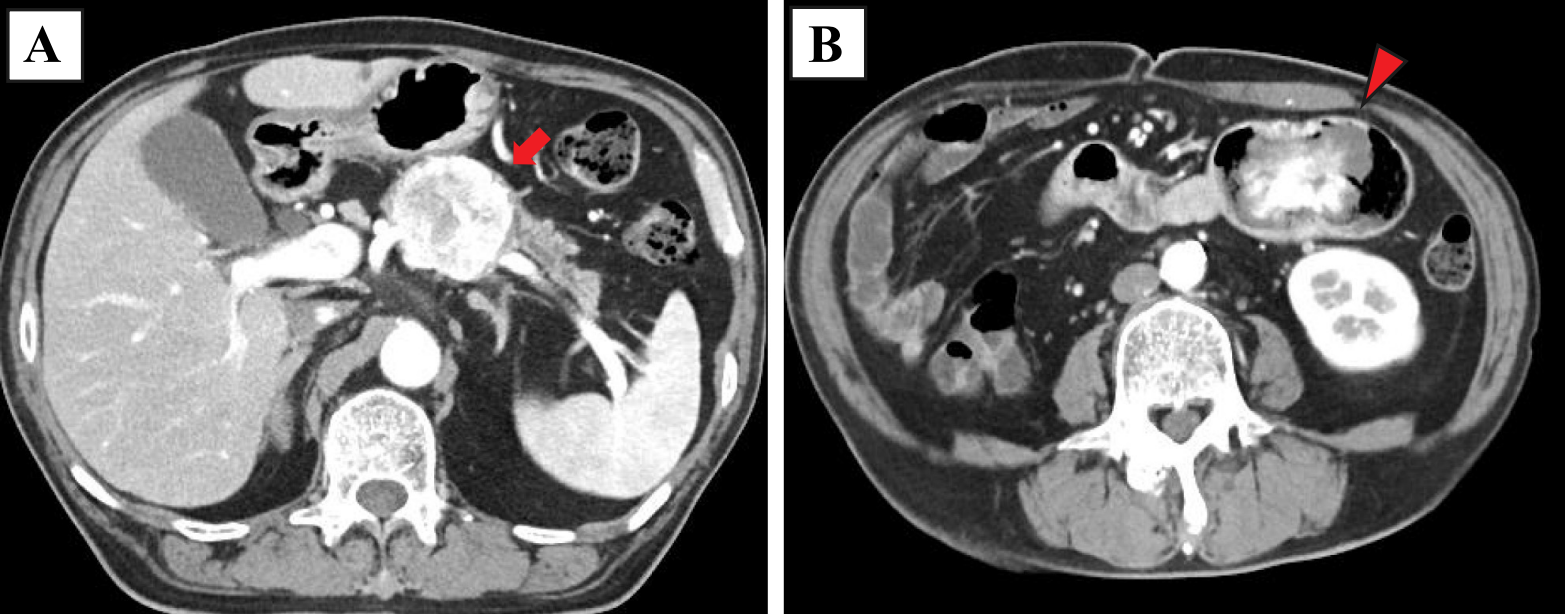
Contrast-enhanced computed tomography findings. (A) Contrast-enhanced computed tomography showing a hypervascular mass (arrow) in the pancreatic body measuring 50 mm in diameter. (B) Axial contrast-enhanced computed tomography image showing a 50-mm hypervascular mass (arrowhead) in the proximal jejunum near the ligament of Treitz.

A 73-year-old man with a history of right nephrectomy for RCC at the age of 32 years was referred to our hospital because of rapidly worsening glycemic control. His medical history included type 2 diabetes mellitus, hypertension, and asthma. On physical examination, he lost 8 kg over the preceding 6 months, and no other significant findings were noted. Laboratory studies revealed a hemoglobin A1c level of 11.0%, with otherwise unremarkable hematologic and biochemical results, except for an elevated carbohydrate antigen 19-9 level of 141.3 U/mL. Contrast-enhanced computed tomography (CT) revealed multiple pulmonary nodules of up to 37 mm in diameter, a 65 mm hypervascular mass in the pancreatic head, and a 50-mm hypervascular mass in the pancreatic body (Fig. 1A). Additionally, a 50-mm hypervascular tumor was identified near the ligament of Treitz, involving the proximal jejunum, without evidence of intestinal obstruction (Fig. 1B). The right kidney had been surgically removed; however, the left kidney appeared normal and was preserved. Based on these findings and the patient’s history, multiple distant metastases of RCC were suspected, although the possibility of neuroendocrine neoplasms could not be excluded. Therefore, endoscopic biopsy was planned for histological confirmation. Esophagogastroduodenoscopy revealed a protruding tumor at the duodenojejunal junction of the small intestine. Although the lesion caused partial luminal narrowing, the endoscope could pass through, allowing for forceps biopsy. Endoscopic ultrasound revealed hypoechoic tumors measuring 56 and 45 mm in the head and body of the pancreas, respectively, and a transgastric fine-needle aspiration was performed for the pancreatic head lesion.

**Figure 2.**
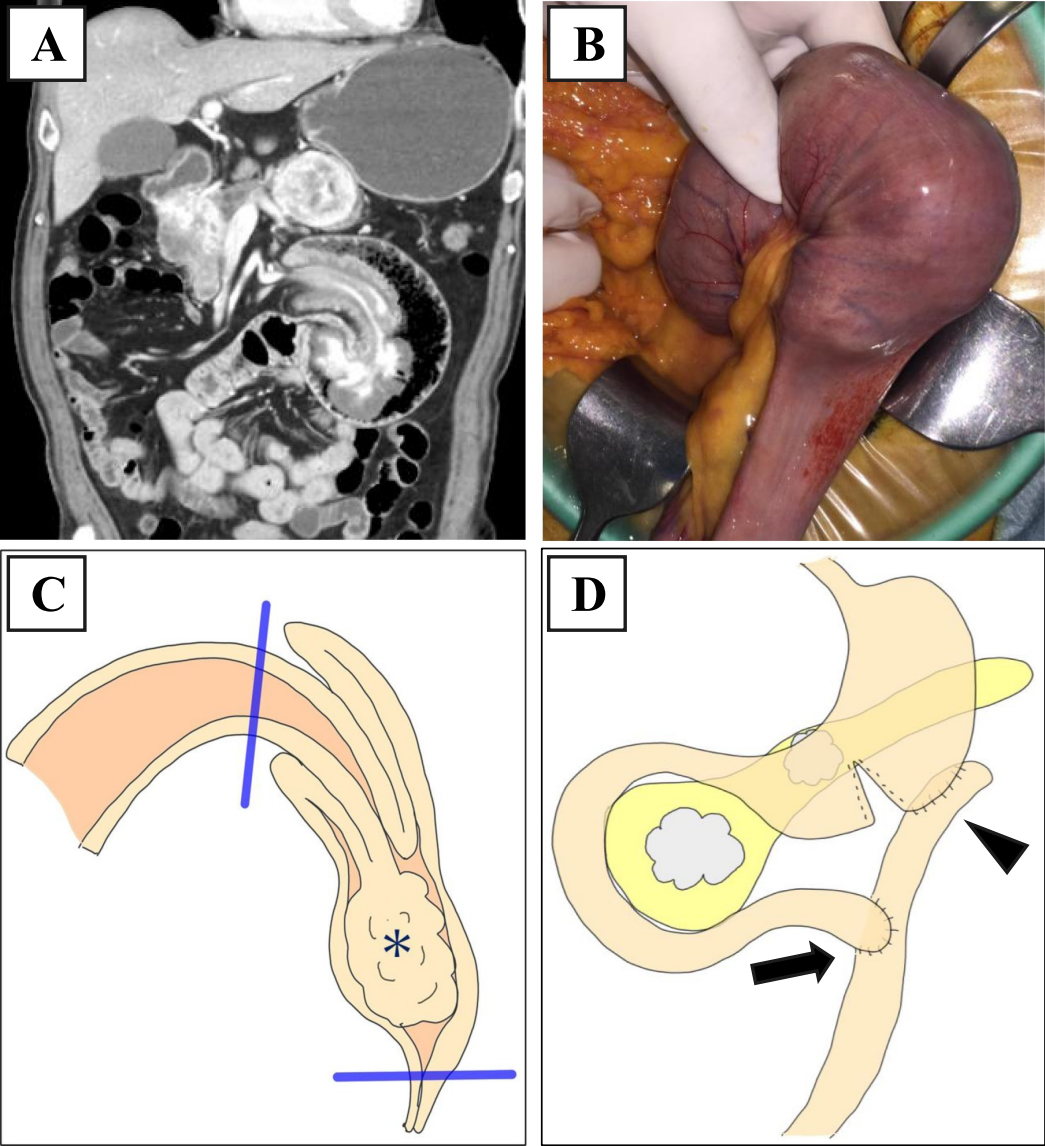
Operative findings. (A) Contrast-enhanced computed tomography demonstrating intussusception in the proximal jejunum. (B) Intraoperative finding showing a tumor originating from the ascending portion of the duodenum and extending into the jejunum. (C) Partial resection of the duodenojejunal junction including the tumor. * Tumor location; blue line indicates the resection line. (D) Bypass reconstruction with gastrojejunostomy (arrowhead) and duodenojejunostomy (arrow).

Five days after the endoscopic biopsy and 7 days after referral, the patient presented to the emergency department with abdominal pain and vomiting. Repeat contrast-enhanced CT revealed jejunal intussusception near the ligament of Treitz, with the known small-bowel tumor as the lead point and proximal bowel dilation (Fig. 2A). Based on the CT findings, the patient was urgently admitted to the hospital. As there were no signs of ischemia or perforation, the obstructive symptoms were intermittent, and the patient remained hemodynamically stable, conservative management with nasogastric decompression was initiated while awaiting definitive pathological diagnosis. However, the patient’s gastrointestinal symptoms persisted without improvement, and biopsy results for both the pancreatic and small-bowel lesions remained inconclusive. Therefore, surgical intervention was performed on day 7 of hospitalization.

Laparotomy revealed a tumor originating from the ascending portion of the duodenum, which caused intussusception and extended approximately 10 cm into the jejunum (Fig. 2B). After opening the omental bursa, a mass was observed in the pancreatic body, with no evidence of serosal invasion or peritoneal dissemination. To obtain a definitive histological diagnosis and determine the appropriate therapeutic strategy, segmental resection of the involved small bowel including the tumor was planned. The ligament of Treitz was divided without attempting manual reduction of the intussusception, and the duodenum was mobilized approximately 3 cm in the oral direction. Partial resection of the duodenojejunal junction, including the tumor arising from the ascending portion of the duodenum, was performed (Fig. 2C). To prevent future gastrointestinal obstruction due to the progression of pancreatic metastases, side-to-side gastrojejunostomy and end-to-side duodenojejunostomy were performed (Fig. 2D). The operation time was 3 hours and 1 minute, with minimal blood loss, and no transfusion was required.

**Figure 3.**
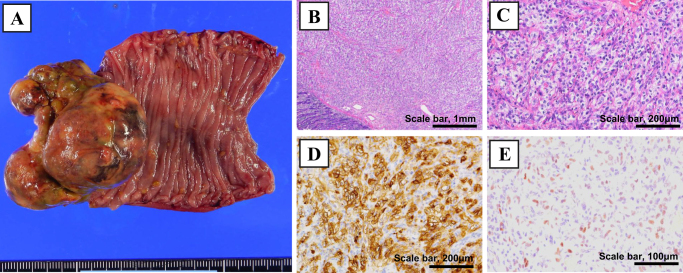
Gross appearance and histopathological findings. (A) Gross appearance. (B) Hematoxylin and eosin (HE) staining, ×4 magnification. (C) HE staining, ×20 magnification. (D) Cluster of differentiation 10 immunostaining: positive, ×20 magnification. (E) Paired box gene 8 immunostaining: focally positive, × 40 magnification.

Gross pathological examination revealed a 50-mm exophytic tumor in the resected specimen (Fig. 3A). Histologically, the tumor consisted of clear cells arranged in solid nests. Immunohistochemical analysis revealed that the tumor was positive for cluster of differentiation 10 and focally positive for paired box gene 8, findings consistent with metastatic RCC (Fig. 3B–E). The postoperative course was uneventful. The patient resumed oral intake on postoperative day 4 and was discharged on day 12. Considering the presence of metastatic RCC, systemic therapy with nivolumab and cabozantinib was initiated according to current European Association of Urology guidelines for metastatic RCC after the patient was discharged[7]. However, the disease progressed, and the patient died 16 months postoperatively.

## Discussion

RCC is characterized by its potential for late recurrence even after curative nephrectomy. Approximately 20–30% of patients experience postoperative recurrence, and about 10% represent late recurrences occurring more than 5 years after surgery^[^1,2,8,9^]^. Hematogenous distant metastasis is the most common pattern of recurrence, frequently involving the lungs, lymph nodes, bones, and liver[4]. Although systemic therapy remains the mainstay of treatment for recurrent disease, current guidelines recommend immune checkpoint inhibitor-based combination therapy, such as nivolumab plus cabozantinib, as a standard first-line treatment option for metastatic RCC^[^7,10^]^. Surgical resection may also be considered when complete removal of metastases is feasible, as it may provide a prognostic benefit^[^7,11^]^. In contrast, small-bowel metastasis from RCC is extremely rare, accounting for approximately 0.7% of all metastatic cases[12].

A PubMed search using the keywords “renal cell carcinoma” and “small bowel metastasis” identified 36 previously reported cases of metachronous small-bowel metastasis following nephrectomy, including the present case (Supplemental Digital Content Table 1, available at: http://links.lww.com/IJSCR/A78)^[^13–45^]^. Most patients were male, with a median age at recurrence of 64 years. The metastatic sites included the duodenum (19%), jejunum (44%), and ileum (39%). The most common presenting symptoms were melena (50%), followed by abdominal pain (39%) and nausea or vomiting (28%). The median tumor size was 41 mm, and intussusception occurred in 31% of cases. Among patients with known metastatic status, 36% had isolated small-bowel metastasis, whereas the remainder had concurrent metastases, most commonly to the lungs. The median interval from nephrectomy to detection of small-bowel metastasis was 5 years. Notably, the present case represents one of the longest recurrence intervals reported in the literature, occurring 41 years after nephrectomy and highlighting the possibility of ultra-late recurrence of RCC even decades after curative surgery.

The small intestine is relatively resistant to hematogenous metastasis, possibly because of factors such as local blood flow dynamics and a well-developed mucosal immune environment^[^5,46,47^]^. Viadana *et al*[48] proposed the concept of “secondary metastasis,” in which RCC metastasizes to the small bowel following initial dissemination to other organs, such as the lungs or liver. However, our review of the 36 cases summarized in Supplemental Digital Content Table 1, available at: http://links.lww.com/IJSCR/A78 revealed that approximately 40% of cases involved isolated small-bowel lesions without concurrent metastases, suggesting that the secondary metastatic pathway may not fully explain all cases. Another characteristic of RCC is tumor dormancy and prolonged latency, which may account for the long interval to recurrence[49]. In the present case, recurrence occurred 41 years after nephrectomy, by which time follow-up had been discontinued and detailed medical records were no longer available. The diagnosis was ultimately confirmed based on histopathological and immunohistochemical findings.

Metastatic small-bowel tumors may cause gastrointestinal bleeding or obstruction, which can compromise oral intake and hinder systemic therapy. Therefore, surgical intervention may be required even when curative resection is not feasible^[^38,44^]^. Our review found that 33 of the 36 patients (92%) underwent surgery. Limited resection is generally feasible for jejunal and ileal metastases unless multifocal disease or extensive ischemia is present[22].

In contrast, when a metastatic lesion is located in the duodenum, careful consideration is required to determine the optimal surgical strategy. Resection may necessitate high-risk procedures such as pancreatoduodenectomy, which carries a substantial risk of serious postoperative complications, including clinically relevant pancreatic fistulas. Such aggressive surgery is generally appropriate only for selected patients with good performance status and favorable oncologic profiles. In other cases, non-surgical management or palliative bypass procedures may be more appropriate. Notably, two of the three nonresected cases in our review^[^29,33,35^]^ involved duodenal metastases.

In the present case, the tumor was located in the ascending portion of the duodenum. Because manual reduction of the intussusception was not possible and a histological diagnosis was required to guide further treatment, partial resection of the duodenojejunal junction was performed. Resection was considered feasible because duodenal mobilization from the ligament of Treitz carried a minimal risk of injuring the uncinate process of the pancreas. Given the large pancreatic metastases and the potential for future gastric or duodenal obstruction, both gastrojejunostomy and duodenojejunostomy were performed. Although long-term survival was not achieved, the patient resumed oral intake and subsequently underwent systemic therapy, which likely contributed to improved quality of life and continuation of systemic therapy.

## Conclusion

This case represents a rare instance of RCC metastasizing to the ascending portion of the duodenum and causing intussusception 41 years after nephrectomy. These findings highlight that RCC may recur even decades after curative surgery and may present with atypical metastatic manifestations such as small-bowel intussusception. Therefore, long-term clinical surveillance and careful evaluation of gastrointestinal symptoms in patients with a remote history of RCC may be important.

## Data Availability

The data supporting the findings of this study are available within the article.
